# Cerebral hypoperfusion, brain structural integrity, and cognitive impairment in older *APOE4* carriers

**DOI:** 10.1007/s11357-025-01642-5

**Published:** 2025-04-12

**Authors:** Ioannis Pappas, Trevor Lohman, Shubir Dutt, Arunima Kapoor, Allison C. Engstrom, John Paul M. Alitin, Samuel Barnes, Ararat Chakhoyan, Lucas Saca, Raghav Gaggar, Elnaz Nourollahimoghadam, Danny J. J. Wang, Mark H. C. Lai, Elizabeth B. Joe, John M. Ringman, Hussein N. Yassine, Lon S. Schneider, Helena C. Chui, Arthur W. Toga, Berislav V. Zlokovic, Daniel A. Nation

**Affiliations:** 1 Laboratory of Neuro Imaging, USC Stevens Neuroimaging and Informatics Institute, Keck School of Medicine, University of Southern California, Los Angeles, CA USA; 2 Leonard Davis School of Gerontology, University of Southern California, Andrus Gerontology Center, 3715 McClintock Ave, Los Angeles, CA 90089 USA; 3 Memory and Aging Center, University of California San Francisco, San Francisco, CA USA; 4 Department of Psychological Science, University of California, Irvine, Irvine, CA USA; 5 Department of Radiology, Loma Linda University, Loma Linda, CA USA; 6 Department of Physiology and Neuroscience, Keck School of Medicine, University of Southern California, Los Angeles, CA USA; 7 Zilkha Neurogenetic Institute, Keck School of Medicine, University of Southern California, Los Angeles, CA USA; 8 Deparment of Psychology, Dana and David Dornsife College of Arts and Letters, University of Southern California, Los Angeles, CA USA; 9 Alzheimer’s Disease Research Center, Keck School of Medicine, University of Southern California, Los Angeles, CA USA; 10 Department of Neurology, Keck School of Medicine, University of Southern California, Los Angeles, CA USA; 11 Department of Psychiatry and Behavioral Sciences, University of Southern California, Los Angeles, CA USA

**Keywords:** Cerebral blood flow, Apolipoprotein E, Brain atrophy, White matter integrity

## Abstract

Cerebral blood flow (CBF) deficits, cognitive decline, and brain structural changes have been reported in older adults with and without apolipoprotein E-e4 (*APOE4*)-related risk for dementia. However, it remains unclear whether brain structural changes mediate the effects of hypoperfusion on cognitive impairment in *APOE4* carriers and non-carriers. We studied 166 (60–89 years) *APOE4* carriers (ε3/ε4 or ε4/ε4) and *APOE3* homozygotes (e3/e3) with and without cognitive impairment by clinical dementia rating (CDR) and neuropsychological testing. Pseudocontinuous arterial spin-labeling-MRI assessed regional CBF, and T1-anatomical and diffusion-MRI assessed structural integrity. Mediation analyses examined relationships among grey matter CBF, grey matter volume, and white matter integrity in regions underlying impairment in distinct cognitive ability domains. *APOE4* carriers with global/memory impairment (CDR 0.5) exhibited decreased CBF in the posterior cingulate, decreased grey matter volume in the hippocampus, parahippocampal gyrus, and posterior cingulate, and decreased white matter integrity in the cingulum relative to *APOE4* carriers with no impairment (CDR 0). Mediation analysis in *APOE4* carriers indicated decreased posterior cingulate CBF effects on global/memory impairment were mediated by decreased cingulum integrity. In the combined *APOE4* and *APOE3* carriers sample, there were direct effects of frontal and inferior parietal CBF and superior longitudinal fasciculus integrity on attention/executive impairment. There were also direct effects of left inferior frontal CBF on language impairment. Findings suggest links between hypoperfusion and brain structural integrity underlying global/memory impairment in *APOE4* carriers. Independent CBF relationships with structural integrity are also identified across genotypes and impairment domains.

## Introduction

Age-related cerebrovascular changes are associated with grey matter atrophy, white matter injury, cognitive decline, and dementia in older adults [1, 2]. In particular, deficits in cerebral blood flow (CBF) occur with aging [3, 4] and may lead to tissue hypoperfusion and a mismatch in neuronal metabolic supply versus demand, possibly contributing to brain injury and neurodegeneration [5]. Consistent with this hypothesis, decreased CBF on perfusion MRI is among the earliest and most predictive biological changes associated with cognitive decline and dementia in older adults [6–8]. The role of cerebral hypoperfusion injury in carriers of the apolipoprotein E-ε4 (*APOE4*) risk gene for Alzheimer’s disease (AD) and dementia is less established.

The *APOE4* allele is the single greatest genetic risk factor for Alzheimer’s disease (AD) and dementia [9, 10], and also conveys increased risk for brain vascular disease [11]. Experiments in humanized *APOE4* transgenic animals suggest that vascular effects of *APOE4* include cerebral hypoperfusion injury [12, 13]. However, research to date in humans suggests a complex pattern of age-related changes in CBF in *APOE4* carriers. For example, some studies in middle-aged and older cognitively unimpaired *APOE4* carriers find increased regional CBF relative to non-carriers [3, 14–16]. It remains unclear whether this early hyperperfusion represents a pathological or compensatory vasodilation, and findings are mixed regarding the association between early CBF differences and better, worse, or equivalent cognition in *APOE4* carriers [3, 15–21]. In contrast, others have reported decreased CBF in older *APOE4* carriers relative to non-carriers [22], and that decreased CBF with aging may be more strongly associated with worsening cognition in *APOE4* carriers relative to non-carriers, particularly in those with cognitive impairment [17, 22–24]. However, not all studies have shown a consistent pattern of perfusion changes in *APOE4* carriers with cognitive impairment [16, 21, 25], and most research has not focused on tying regional hypoperfusion patterns to structural changes underlying specific cognitive ability domains.

One study focusing specifically on memory regions found decreased CBF in older *APOE4* carriers with memory impairment [24], a finding that was particularly pronounced when evidence of structural volume loss was also present in those memory regions [23]. These findings could suggest that age-related deficits in CBF may impact cognition through changes in brain structural integrity in *APOE4* carriers. We hypothesize that age-related decline in CBF in *APOE4* carriers may cause cognitive impairment through hypoperfusion injury to brain grey and white matter structures underlying important cognitive ability domains. To address this question, the present study investigates regional CBF differences between older *APOE4* carriers and *APOE3* homozygotes with and without cognitive domain impairment, and whether differences in related grey and white matter structural integrity mediate the effects of hypoperfusion on cognitive impairment in relevant ability domains.

## Methods

### Participants

All 166 participants were recruited from the community and the USC Alzheimer’s Disease Research Center (ADRC) if they were living independently and between the ages of 55 and 89. Study exclusions were a history of clinical stroke, dementia, moderate-to-severe traumatic brain injury, major psychiatric or neurologic illness, substance abuse, organ failure or major systemic illness, or active medications or other medical conditions that could impact cognitive function, as well as contraindications for contrast-enhanced brain MRI. Participants were not excluded based on the presence of stable, controlled cardiovascular risk factors, such as hypertension, hyperlipidemia, coronary artery disease, or type 2 diabetes. The study and procedures were approved by the USC ADRC Institutional Review Board indicating compliance with all ethical regulations. Informed consent was obtained from all participants before study enrolment.

### Brain MRI

All MRI scans were acquired using 3 T scanner at Mark and Mary Stevens Neuroimaging and Informatics Institute of USC (Siemens Prisma 32-channel head receive coil and body transmit coil scanner). All 166 participants underwent a high-resolution 3D T1-weighted magnetization-prepared rapid acquisition gradient echo (MPRAGE) MRI T1-weighted for structural imaging with the following parameters: TR/TE = 7.37/3.05 ms; TI = 400 ms; NEX = 1; flip angle = 11°; FOV = 256 × 256 mm with isotropic 1-mm voxel size; and 196 slices. T1-weighted image denoising, bias correction, and brain mask extraction were performed using the Rican correction method [26], N4BiasFieldCorrection command [27], and antsBrainExtraction.sh command [28] respectively. The T1 image was registered to the *b* = 0 s/mm^2^ and the fractional anisotropy (FA) images [29] using nonlinear Syn ANTs multivariate option with two target images (b0 and FA) and the moving T1 image. The white matter, grey matter, and cerebrospinal fluid partial volume maps were extracted with *fast* from the FSL package [30].

#### Perfusion MRI

All 166 participants underwent cerebral perfusion MRI with a 3D gradient and spin-echo pseudo-continuous arterial spin labeling (pCASL) sequence with background suppression. Parameters were as follows: 2.5 mm^3^ isotropic resolution, matrix 96 × 96, 48 slices, 4 segments, TR/TE = 4300 ms/36.8 ms, 120° flip angle, label duration = 1500 ms, post-labeling delay = 2000 ms, labeling efficiency = 0.73. The pCASL scans were pre-processed using the ASLtbx pipeline, implemented in SPM12 within MATLAB [31, 32]. Pre-processing steps for pCASL scans included [33] motion correction, co-registration to individual subject’s structural T1-weighted image, spatial smoothing with a 6-mm full-width at half-maximum Gaussian kernel, and tag-control subtraction resulting in 15 tag-control pairs for each subject with values for absolute CBF (mL/100 g tissue/min). All CBF images were thresholded below 10 or above 150 mL/100 g/min to exclude CBF outside the expected physiological range of grey matter [33, 34]. Tag-control pairs were warped to MNI space and averaged to create mean CBF maps for each subject. Resulting mean CBF maps were visually inspected for quality and gross abnormalities (i.e., large signal dropout). Partial volume correction was performed by applying subject-specific grey matter masks derived from the grey matter tissue class segmentation of T1-weighted structural images [35]. Segmented grey matter maps were thresholded at 0.3, binarized, and multiplied by the mean CBF maps to ensure CBF was limited to grey matter. Regional CBF values were extracted from all ROIs using the AAL3 atlas and were normalized by residualizing to the precentral gyrus using linear regression analysis. All CBF data are reported as standardized residual *z* scores.

#### Diffusion MRI

A subset of 99 participants from the overall sample of 166 undergoing perfusion MRI also underwent high-angular diffusion tensor imaging (DTI) to acquire diffusion weighted images (DWI) with a spin-echo EPI sequence with the following parameters: TR/TE = 3230/89.2 ms, flip angle = 78°, matrix = 140 × 140, 92 slices of 1.5 mm with 1.25 × 1.25-mm voxel size, multiband factor = 4, echo spacing = 0.78 ms, BW = 1700 Hz/Px, partial Fourier factor 0.75. After post-processing and quality control, 96 out of the 99 participants undergoing DTI-MRI had diffusion images of sufficient quality for analysis. First, DWI were denoised based on the dwidenoise from MRtrix3 using the MP-PCA method [36]. For Eddy/Topup, a bet prelim DWI task is first extracted using FSL’s *bet* method [37] and Eddy/Topup was run only within the mask to correct the brain deformation induced by the magnetic field susceptibility artifacts. Next, *eddy* was used to correct eddy-currents, motion artifacts and to perform slice-wise outlier detection and correction. Phase-encoding direction was AP, and a reverse phase encoded *b* = 0 s/mm^2^ image was provided by using an additional PA image. The *topup* command was run on the *b* = 0 s/mm^2^ and reversed phase encoded *b* = 0 s/mm^2^ images to extract the deformation field. The deformation field was applied, and the *eddy* command was performed using the *topup* output. A brain extraction mask was extracted from the *b* = 0 s/mm^2^ image (using *bet*) and applied to the whole brain DWI. An N4 bias field correction was performed on the *b* = 0 s/mm^2^ image using the ANTs command *N4BiasFieldCorrection*. The DWI was cropped to reduce the bounding box and resampled to 1 mm to match the T1 resolution. Diffusion reconstruction occurred using *b* values of 1500 s/mm^2^. The metrics computed from the diffusion tensor reconstruction [38, 39].

White matter tractography analysis was conducted with DWI images processed using the TractoFlow pipeline [40] that uses Nextflow [41] and singularity [42]. The pipeline computes DTI maps, and a whole brain tractogram and calls different functions from various neuroimaging software packages, namely FSL [43], MRtrix3 [44], ANTs 28, and DIPY [45]. White matter bundles were extracted using the RecoBundles framework as implemented in DIPY [45]. RecoBundles transforms the tractogram to MNI space using streamline-based linear registration then extracts the tracts from the registered tractogram using the MNI atlas “Advanced_Atlas_of_80_Bundles_in_MNI_space” (https://figshare.com/articles/dataset/Advanced_Atlas_of_80_Bundles_in_MNI_space/7375883) and extracts the uncinate fasciculus (UF) (left and right), the cingulum (left and right), and the superior longitudinal fasciculus (SLF) tracts. Extracted FA values of segments along the tracts were used in statistical analysis. After quality control analysis of processed DTI data, the pipeline failed to provide any usable data on three participants, yielding a subset of *n* = 96 with usable DTI data for cingulum and SLF tracts, and a subset of *n* = 94 with usable UF tract data.

### APOE genotyping

Briefly, DNA was extracted from buffy coat followed by *APOE* genotyping by polymerase chain reaction (PCR)-restriction fragment length approach [46]. Participants were stratified based on *APOE* genotype as *APOE4* carriers (ε3/ε4 and ε4/ε4) and homozygote *APOE3* allele (ε3/ε3).

### Cognitive and neuropsychological evaluation

Each participant underwent clinical dementia rating (CDR) evaluation and standardized clinical interview as part of health history assessment, a physical examination, and neuropsychological testing according to unified data set (UDS) (version 2.0/3.0) procedures. For global/memory domain impairment, impairment was defined as CDR 0.5 and unimpaired as CDR 0. For attention/executive and language domain impairments, we applied previously validated neuropsychological criteria for cognitive domain impairment versus cognitively unimpaired [47]. For attention/executive domain impairment, impairment was defined as having two or more attention/executive domain test scores falling > 1 standard deviation below demographically-corrected normative values. Attention/executive test scores included Trails A, Trails B, and digit/number span. For language domain impairment, impairment was defined as having two or more language domain test scores falling > 1 standard deviation below demographically corrected normative values. Language domain test scores included animals, F-A-S, and confrontation naming (Multilingual Naming Test/Boston Naming Test). All normative values were corrected for age, sex, and education using publicly available UDS version2/3-derived regression equations.

### Vascular risk factors (VRFs)

Each participant underwent a physical examination, blood tests, and interviews to determine presence of VRFs. The presence of VRFs were defined based on classification from the Framingham Stroke Risk Profile and included a history of cardiovascular disease (heart failure, angina, stent placement, coronary artery bypass graft, intermittent claudication), hypertension, hyperlipidemia, type 2 diabetes, atrial fibrillation, and transient ischemic attack or minor stroke. The total burden was defined by the sum of these specific risk factors. Based on prior studies linking 2 + VRFs vs. 0–1 VRFs to cerebrovascular pathology [48, 49], participants were grouped by total VRF burden of 0–1 vs. 2 +.

### Statistical analysis

All study variables were screened for outliers through visual inspection of variable distributions and removal of influential outliers greater than or equal to +/− 3 standard deviations from the overall sample mean. This led to removal of 1 hippocampal CBF value, 1 parahippocampal gyrus value, 1 uncinate fasciculus FA value, 1 cingulum FA value, and 1 superior longitudinal fasciculus value. Based on prior studies [23, 24], our a priori hypothesis was that cognitively unimpaired *APOE4* carriers would exhibit decreased CBF relative to *APOE3* homozygotes, and cognitively impaired *APOE4* carriers would exhibit decreased CBF relative to cognitively unimpaired *APOE4* carriers, and that *APOE4* carriers with cognitive impairment would show decreased CBF relative to cognitively impaired *APOE3* homozygotes. This hypothesis drove our a priori planned comparisons of *APOE4* carriers versus *APOE3* homozygotes with and without cognitive impairment in regions involved in memory and global cognitive function, including the hippocampal, parahippocampal and posterior cingulate regions. Pairwise group comparisons from a 2 × 2 analysis of covariance (ANCOVA), comparing *APOE* status (*APOE4* vs. *APOE3/3*) × cognitive status (CDR 0.5 vs. 0), controlling for age, sex, education, and VRF burden (2 + vs. 0–1 VRFs) were utilized as previously validated [48, 49]. Findings were also confirmed by hierarchical logistic regression analysis of regional CBF as a predictor of cognitive status (CDR 0.5 vs. 0) with same covariates.

To determine whether cerebrovascular changes may be related to changes in grey and white matter structural integrity underpinning memory and global cognitive function, the same approach was applied to evaluation of group differences (2 × 2 ANCOVA: *APOE* status × cognitive status) in hippocampal and parahippocampal volume, as well as white matter integrity (DTI FA values) in the connected uncinate fasciculus. Posterior cingulate gyrus volume and white matter integrity in the connected cingulum tract were also evaluated. All analyses controlled for age, sex, education, and VRF burden. For brain regions showing differences in CBF or structural integrity related to genotype and/or cognitive impairment, mediation analysis was used to confirm direct effects of regional CBF independent of brain structural integrity and to test whether indirect effects of regional CBF were mediated by brain structural integrity on cognitive status (CDR 0.5 vs. 0), controlling for age, sex, education, and VRF burden. Exploratory analyses using the same approach examined CBF, and grey and white matter integrity in connected regions and tracts underpinning attention/executive and language domains, and both main effects and pairwise comparisons were explored. Attention/executive and language domain impairments were age-, sex-, and education-corrected, and all models additionally corrected for VRF burden.

To investigate regional FA differences along the white matter tracts, we used the BUndle ANalytics (BUAN) [50] framework alongside custom scripts. Briefly, the FA values for all segments of the tracts were extracted as an.h5 object. These values were then used as predicted values in a linear mixed effects model with group, age, sex, education, and vascular risk factors as predictors. Group was a variable representing group membership during the comparison between the respective groups (for example *APOE4* vs. *APOE3*). A *p* value per segment was obtained (uncorrected) for each tract. *p* values < 0.05 were plotted as red segments over the respective tract. Mean FA values over the entire tracts were also extracted and used in the *swarmplot* figures.

All analyses were 2-tailed with significance set at *p* < 0.05. False discovery rate (FDR)-correction (Benjamini-Hochberg) was applied for all a priori planned comparisons to address multiple comparisons. Follow-up confirmatory, exploratory, and BUAN tract segment analyses were not multiple comparisons corrected. Mediation analysis using the Hayes process macro in R findings are presented as the regression coefficient (unstandardized β) with Bootstrap 95% confidence interval (CI). Statistical analyses were performed in R and SPSSv29.

## Results

**Fig. 1:**
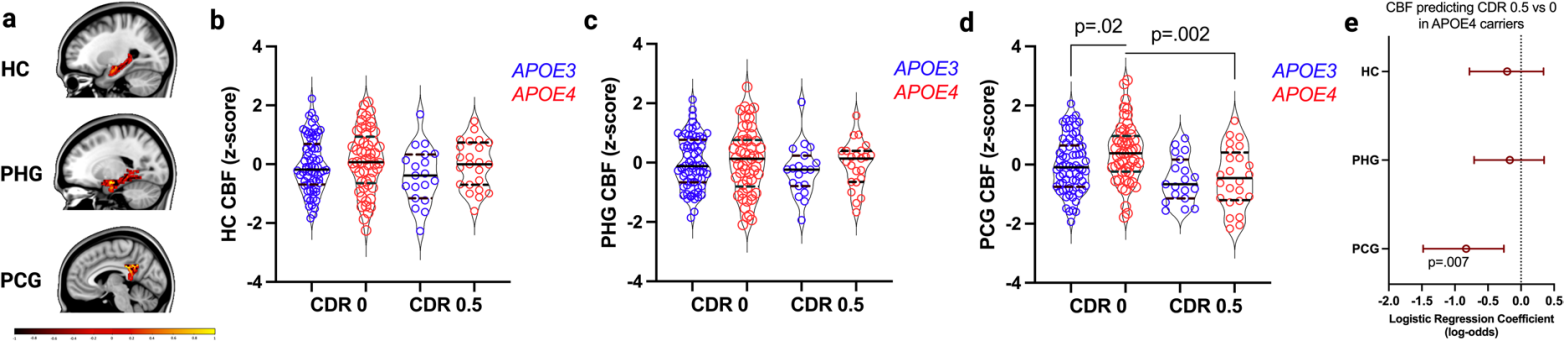
**a** Representative CBF images from ROIs related to global/memory impairment are displayed, including hippocampus (HC), parahippocampal gyrus (PHG), and posterior cingulate gyrus (PCG). Scale bar is − 1 to + 2 residualized *z* score. **b**–**d** Regional CBF values (residualized *z* scores) are displayed in violin plots with median and interquartile range and compared by *APOE4* status (*APOE3* homozygotes [blue] vs. *APOE4* carriers [red]) and global/memory impairment by clinical dementia rating (CDR) global score (0 vs. 0.5): CDR 0 *APOE3* (*n* = 70), CDR 0 *APOE4* (*n* = 53), CDR 0.5 *APOE3* (*n* = 19), CDR 0.5 *APOE4* (*n* = 24). **e** Findings from confirmatory hierarchical logistic regression analysis are shown with logistic regression coefficients displayed on the *x* axis expressed in log-odds units, and error bars representing standard errors. All analyses controlled for age, sex, education, and VRF burden

Demographic and clinical characteristics of the sample are displayed in Table 1. For regions underlying global/memory function, no CBF differences by genotype or CDR status were observed in the hippocampus or parahippocampal gyrus. In the posterior cingulate gyrus, global/memory impaired *APOE4* carriers exhibited significantly decreased CBF relative to unimpaired *APOE4* carriers, *p* = 0.002. Additionally, unimpaired *APOE4* carriers showed a small but statistically significant increase in CBF in the posterior cingulate gyrus relative to unimpaired *APOE3* homozygotes (*p* = 0.02); however, this finding did not survive FDR correction for multiple comparisons (Fig. 1a–d). Results in *APOE4* carriers were confirmed by logistic regression analysis showing only posterior cingulate CBF predicts global/memory impairment by CDR, *p* = 0.007 (Fig. 1e). All models controlled for age, sex, education, and VRF burden.

**Table 1:** Demographic and clinical characteristics of the sample

*APOE* genotype		*APOE3/3*	*APOE3/4*	*APOE3/3*	*APOE3/4*
CDR score		0	0	0.5	0.5
pCASL-MRI *N* = 166	*n*	70	53	19	24
MPRAGE-MRI *N* = 166	*n*	70	53	19	24
DTI-MRI (Cingulum) Subset of *n* = 95	*n*	42	34	8	11
DTI-MRI (UF) Subset of *n* = 93	*n*	41	33	8	11
DTI-MRI (SLF) Subset of *n* = 95	*n*	42	34	8	11
Age, years	Mean (SD)	69.8 (6.8)	68.2 (7.3)	72.0 (8.1)	71.5 (6.7)
Female	*n* (%)	29 (41.4)	21 (39.6)	10 (52.6)	11 (45.8)
Education, years	Mean (SD)	16.6 (2.4)	16.3 (2.1)	15.4 (3.3)	15.9 (2.5)
Attention/executive impairment	*n* 0/1	49/5	38/4	9/2	6/5
Language impairment	*n* 0/1	50/5	38/5	10/2	7/9
Vascular risk factor burden	*n* 0–1/2 +	41/29	27/26	9/10	11/13

Of the *N* = 166 with pCASL-MRI data, a subset of *n* = 99 also underwent diffusion imaging and *n* = 96 of these participants had usable cingulum and SLF data, and *n* = 93 had usable UF data. One outlier was then removed from each tract to yield final numbers displayed. Numbers for the participant subset in the final analysis are presented above after outlier removal (see Methods for details). *CDR*, clinical dementia rating; *pCASL*, pseudocontinuous arterial spin-labeling; DTI, diffusion tensor imaging; *MRI*, magnetic resonance imaging; *SD*, standard deviation; *UF*, uncinate fasciculus; *SLF*, superior longitudinal fasciculus

**Fig. 2:**
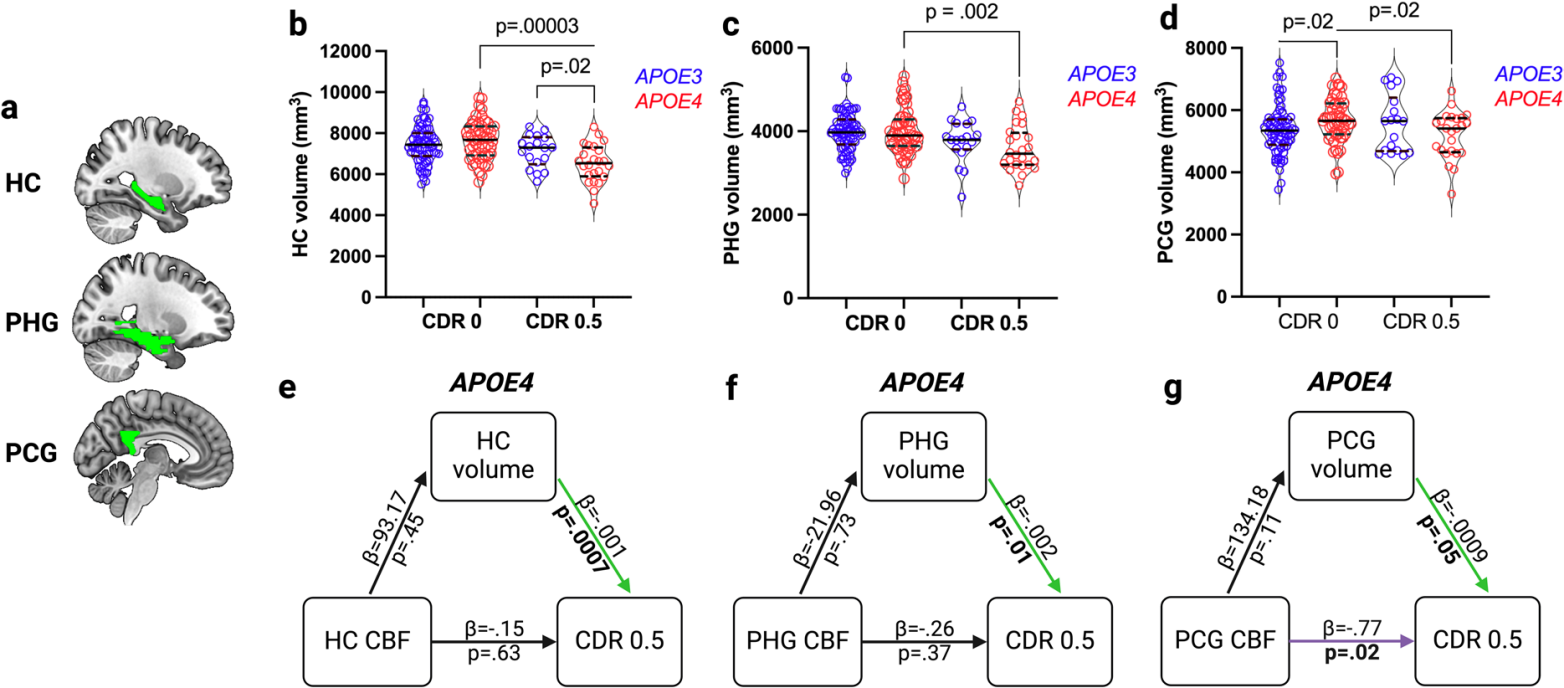
**a** Volumetric ROIs are displayed for regions implicated in global/memory impairment, including hippocampus (HC), parahippocampal gyrus (PHG), and posterior cingulate gyrus (PCG). **b**–**d** Regional volumes are shown in comparisons of *APOE4* status (*APOE3* homozygotes [blue] vs. *APOE4* carriers [red]) by global/memory impairment by clinical dementia rating (CDR) global score (0 vs. 0.5): CDR 0 *APOE3* (*n* = 70), CDR 0 *APOE4* (*n* = 53), CDR 0.5 *APOE3* (*n* = 19), CDR 0.5 *APOE4* (*n* = 24). **e**–**g** Results of mediation analysis are displayed for *APOE4* carriers. *Purple line* indicates significant direct effect of CBF on global/memory impairment in PCG independent of regional volume, and *green lines* indicate significant effects of volume reduction on global/memory impairment in HC and PHG, independent of regional CBF. All analyses controlled for age, sex, education, and VRF burden

For structural grey matter measures in global/memory regions, we observed significantly decreased hippocampal (*p* = 0.00003), parahippocampal (*p* = 0.002), and posterior cingulate (*p* = 0.02) volumes in global/memory impaired *APOE4* carriers relative to unimpaired *APOE4* carriers (Fig. 2a–d). Additionally, global/memory impaired *APOE4* carriers also showed slightly decreased hippocampal volume relative to impaired *APOE3* homozygotes (*p* = 0.02), and unimpaired *APOE4* carriers had slightly greater posterior cingulate volume than unimpaired *APOE3* homozygotes (*p* = 0.02); however, these smaller effects did not survive FDR correction for multiple comparisons. Mediation analyses confirmed effects of hippocampal (*p* = 0.0007) and parahippocampal (*p* = 0.01) volume reduction on global/memory impairment, independent of regional CBF (Fig. 2e–f). In the posterior cingulate, regional CBF had a direct effect on global/memory impairment independent of volume reduction (*p* = 0.02), and volume reduction showed a nonsignificant trend (*p* = 0.05) towards an effect on global/memory impairment independent of regional CBF (Fig. 2g). No significant indirect mediation effects were observed. All models controlled for age, sex, education, and VRF burden.

**Fig. 3:**
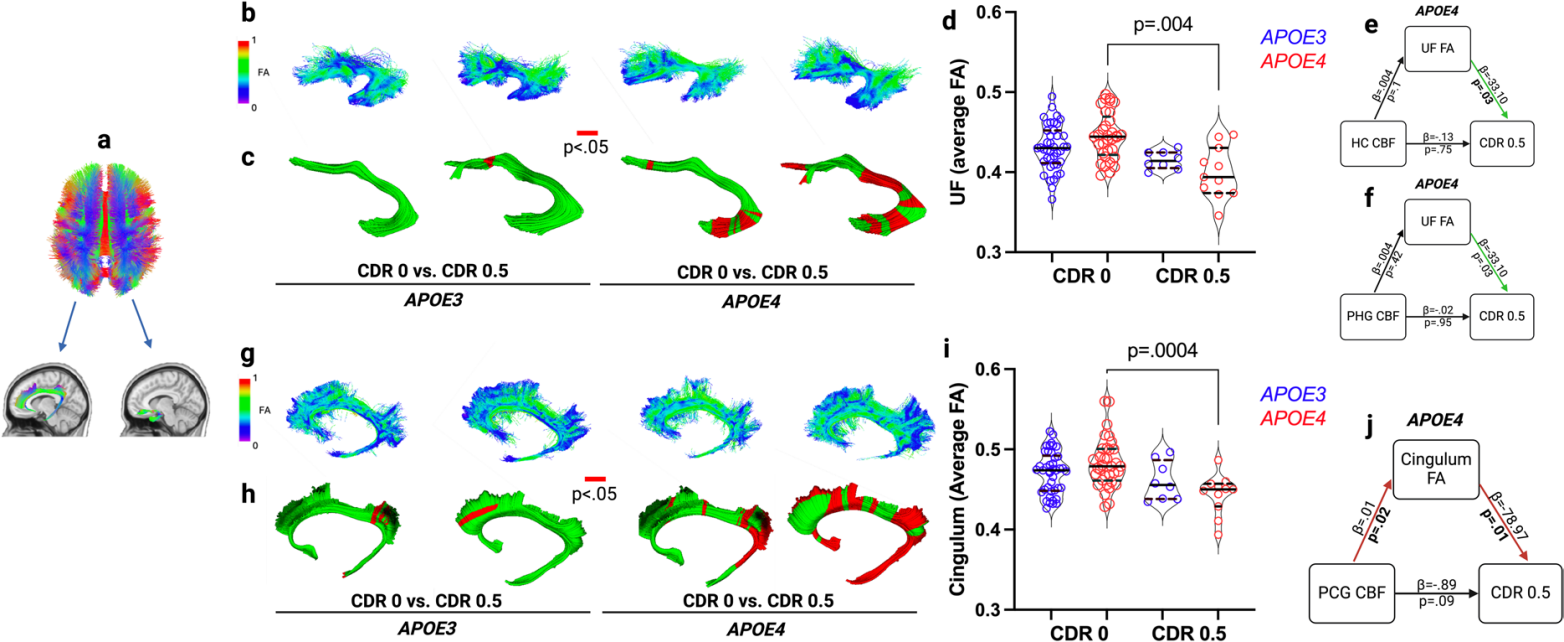
**a** White matter tract fractional anisotropy (FA) maps of the uncinate fasciculus (UF) and cingulum tracts connected to grey matter regions supporting global/memory functions. **b** Examples of individual UF tracts for each group. **c** UF tract segment-level differences in (left and right) UF FA between groups are displayed with red segments representing areas with significantly (*p* <.05) lower FA in CDR 0.5 vs. 0 by *APOE* carrier status. **d** UF FA values averaged over the entire tract and across left and right tracts are displayed in violin plots with median and interquartile range and compared by *APOE* status (*APOE3* homozygotes [blue] vs. *APOE4* carriers [red]) and global/memory impairment by clinical dementia rating (CDR) global score (0 vs. 0.5): CDR 0 *APOE3* (*n* = 41), CDR 0 *APOE4* (*n* = 34), and for CDR 0.5 *APOE3* (*n* = 8), CDR 0.5 *APOE4* (*n* = 11). **e–f** Mediation analysis for *APOE4* carriers with green lines indicating significant effects of UF FA, independent of CBF in connected hippocampal (HC) and parahippcampal (PHC) grey matter. **g** Examples of individual cingulum tracts. **h** Segment-level differences in cingulum FA. **i** Cingulum FA values compared by group: CDR 0 *APOE3* (*n* = 42) *APOE4* (*n* = 34), CDR 0.5 *APOE3* (*n* = 8) *APOE4* (*n* = 11). **j** Mediation analysis for *APOE4* carriers with red lines indicating posterior cingulate gyrus (PCG) CBF effects mediated by Cingulum FA. All analyses controlled for age, sex, education, and VRF burden

Structural integrity of the uncinate fasciculus white matter tract connected to hippocampal and parahippocamal global/memory regions showed decreased integrity (DTI FA) in global/memory impaired *APOE4* carriers relative to unimpaired *APOE4* carriers (*p* = 0.003), and segment-level analysis showed significant differences in uncinate fasciculus segments more proximal to hippocampal and parahippocampal regions (Fig. 3a–d). Mediation analysis confirmed the effect of uncinate fasciculus integrity on global/memory impairment in *APOE4* carriers, independent of regional CBF in the hippocampus (*p* = 0.02) and parahippocampal gyrus (*p* = 0.02) (**Fig. e–f**). Structural integrity of the cingulum white matter tract connected to the posterior cingulate global/memory region showed decreased integrity in global/memory impaired *APOE4* carriers relative to unimpaired *APOE4* carriers (*p* = 0.0004), and segment-level analysis showed significant differences in cingulum segments more proximal to the posterior cingulate and hippocampal and parahippocampal regions (Fig. 3g–i). Mediation analysis revealed that the effect of posterior cingulate CBF on global/memory impairment in *APOE4* carriers was not direct but was mediated by decreased white matter integrity in the connecting cingulum tract, indirect effect *β* = − 0.7936, 95% CI (− 124.1293, − 0.1541) (Fig. 3g–j). All models controlled for age, sex, education, and VRF burden.

**Fig. 4:**
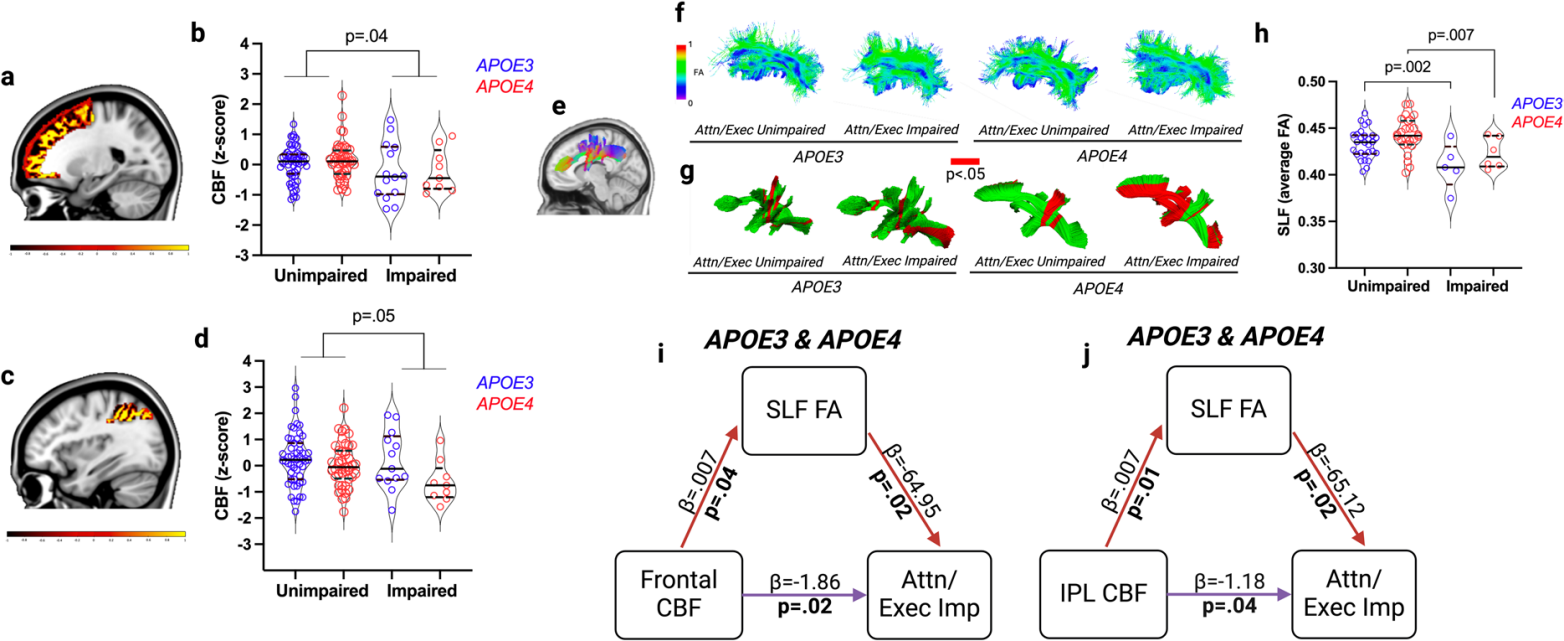
**a** Representative frontal lobe (superior, middle, and inferior frontal gyri) CBF map. Scale bar is − 1 to + 2 residualized *z* score. **b** Regional frontal lobe CBF compared by group *APOE* status (*APOE3* homozygotes [blue] vs. *APOE4* carriers [red]) and attention/executive impairment: unimpaired *APOE3* (*n* = 51), unimpaired *APOE4* (*n* = 44), impaired *APOE3* (*n* = 14), impaired *APOE4* (*n* = 9). **c** Representative inferior parietal lobe (IPL) CBF map. Scale bar is − 1 to + 2 residualized *z* score. **d** Regional frontal lobe CBF values compared by group: unimpaired *APOE3* (*n* = 51), unimpaired *APOE4* (*n* = 44), impaired *APOE3* (*n* = 14), impaired *APOE4* (*n* = 9). **e** Fractional anisotropy (FA) maps of the superior longitudinal fasciculus (SLF) connecting frontal and IPL grey matter regions supporting attention/executive functions. **f** Examples of individual SLF tracts. **g** Segment-level tract (left and right) differences in SLF FA between groups are shown in red. **h** SLF FA values averaged (left and right) along the entire tract and compared by group: unimpaired *APOE3* (*n* = 28), unimpaired *APOE4* (*n* = 26), impaired *APOE3* (*n* = 5), unimpaired *APOE4* (*n* = 6). **i** Mediation analysis for combined sample of *APOE3* homozygotes and *APOE4* carriers with purple lines indicating significant direct effects of CBF, independent of SLF FA, and red lines indicate significant CBF effects on SLF FA and SLF FA effects on attention/executive impairment. **j** Mediation analysis for combined sample of *APOE3* homozygotes and *APOE4* carriers with purple lines indicating significant direct effects of CBF, independent of SLF FA, and red lines indicate significant CBF effects on SLF FA and SLF FA effects on attention/executive impairment. All analyses controlled for age, sex, education, and VRF burden

Exploratory analysis of CBF and structural integrity in regions underlying impairment in attention/executive and language domains identified effects in the combined sample of *APOE4* carriers and *APOE3* homozygotes. First, we analyzed brain structures underpinning attention/executive abilities, including frontal and inferior parietal CBF and structural volume, and white matter structural integrity in the superior longitudinal fasciculus tract that connects frontal and inferior parietal regions. Participants with attention/executive impairment in the combined sample exhibited decreased frontal CBF (*p* = 0.04) and a trend (*p* = 0.05) towards a significantly decreased inferior parietal CBF relative to those who were unimpaired (Fig. 4a–d). Structural analysis indicated no differences in grey matter volume within these structures (data not shown), but both *APOE4* carriers and *APOE3* homozygotes with attention/executive impairment exhibited decreased white matter integrity in the superior longitudinal fasciculus tract that connects frontal and parietal regions relative to *APOE4* carriers and *APOE3* homozygotes who were unimpaired, and segment level analysis showed varied distribution of significant differences along the tract (Fig. 4e–h). Mediation analysis confirmed direct effects of frontal CBF (*p* = 0.02) and inferior parietal CBF (*p* = 0.04) on attention/executive impairment, independent of superior longitudinal fasciculus integrity. In addition to these independent effects of CBF, there were effects of frontal CBF on superior longitudinal fasciculus integrity (*p* = 0.04), effects of superior longitudinal fasciculus integrity on attention/executive impairment (*p* = 0.02), effects of inferior parietal CBF on superior longitudinal fasciculus integrity (*p* = 0.01), and effects of superior longitudinal fasciculus integrity on attention/executive impairment, *p* = 0.02 (Fig. 4i–j). However, neither of the indirect effects from the mediation analyses were statistically significant: frontal CBF, *β* = − 0.4458, 95% CI (− 1.7433, 0.0615); inferior parietal CBF, *β* = − 0.4345, 95% CI (− 1.7954, 0.0282).

**Fig. 5:**
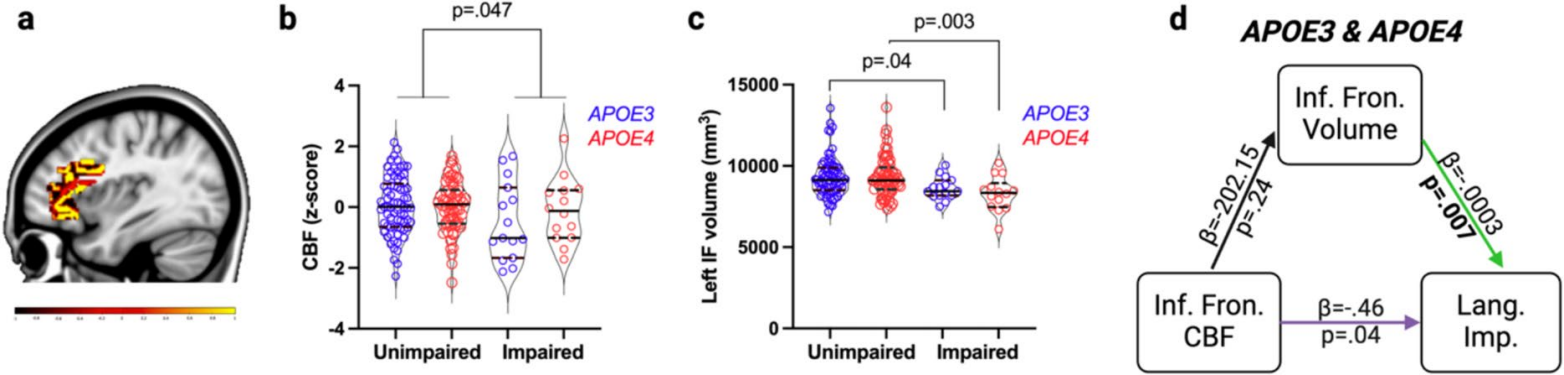
**a** Representative left inferior frontal lobe CBF map. Scale bar is − 1 to + 2 residualized *z* score. **b** Regional left inferior frontal lobe CBF values and **c** volumes compared by *APOE* status (*APOE3* homozygotes [blue] vs. *APOE4* carriers [red]) and language impairment: unimpaired *APOE3* (*n* = 52), unimpaired *APOE4* (*n* = 45), impaired *APOE3* (*n* = 15), impaired *APOE4* (*n* = 14). **d** Mediation analysis for the combined sample of *APOE3* and *APOE4* carriers with purple line indicating significant direct effect of CBF, independent of regional volume, and green line indicating significant effect of volume reduction, independent of regional CBF. All models controlled for age, sex, education, and VRF burden

Finally, we analyzed regional CBF and structural volume in left inferior frontal regions involved in language ability. Participants with language impairment in the overall sample of combined *APOE4* carriers and *APOE3* homozygotes exhibited a trend towards decreased left inferior frontal CBF (*p* = 0.047), and both *APOE4* carriers (*p* = 0.003) and *APOE3* homozygotes (*p* = 0.04) exhibited decreased grey matter volume, relative to those who were unimpaired (Fig. 5a–d). Mediation analysis confirmed direct effects of left inferior frontal CBF on language impairment independent of inferior frontal volume (*p* = 0.04), as well as effects of inferior frontal volume independent of inferior frontal CBF, *p* = 0.007 (Fig. 5d).

## Discussion

Here we report a pattern of regional CBF and structural differences in cognitively impaired relative to unimpaired *APOE4* carriers and *APOE3* homozygotes, with both independent and structurally mediated CBF effects on different cognitive ability domains. The findings overall suggest that *APOE4* carriers display a distinct pattern of regional CBF that is characterized by posterior cingulate gyrus hypoperfusion that is related to impairment in memory and global cognition through decreased cingulum integrity. These findings are consistent with prior studies implicating posterior cingulate hypoperfusion in cognitive impairment [51, 52], and reveal an apparent susceptibility of *APOE4* carriers to hypoperfusion in this region. Findings further suggest that the contribution of posterior cingulate hypoperfusion to cognitive impairment may be related to injury of the connected cingulum white matter, particularly in areas proximal to connections of the posterior cingulate gyrus and medial temporal lobe. Together the findings are consistent with the established susceptibility of white matter to hypoperfusion [53], the importance of white matter injury in vascular contributions to cognitive impairment [54], and preclinical studies demonstrating *APOE4* causes white matter injury and cognitive impairment through neurovascular dysfunction [13]. These findings have major clinical significance given *APOE4* carriers with global/memory impairment are at very high risk for progression to dementia [55].

We did not find any differences in hippocampal or parahippocampal CBF in relation to *APOE4* status or cognitive status, but we did find an expected decrease in the volume of these brain structures in *APOE4* carriers with global/memory impairment. Importantly, these volume differences were found to have statistical effects on global/memory impairment that were independent of regional CBF, potentially suggesting that neurodegeneration within the hippocampus and parahippocampal gyrus in *APOE4* carriers is not driven by hypoperfusion injury. This is consistent with prior perfusion imaging studies focusing on these memory regions in early-stage cognitive impairment, which have shown mixed results, including increased, decreased, and no changes relative to unimpaired controls [16, 21, 25, 51, 52]. We also observed both decreased CBF and decreased volume in the posterior cingulate in *APOE4* carriers specifically, but the statistical effects of posterior cingulate CBF and volume on global/memory impairment were independent. This could suggest distinct effects of hypoperfusion and neurodegeneration on global/memory impairment in *APOE4* carriers.

The dominant interpretation of posterior cingulate hypoperfusion in older adults with cognitive impairment is that it represents secondary effects of neurodegeneration and decreased neuronal metabolism [56]. This hypothesis is supported by studies showing strong correlations between perfusion MRI and fluorodeoxyglucose-positron emission tomography (FDG-PET) [57]. However, it has also been proposed that the hypoperfusion of this region could be due to vascular dysfunction and may contribute to cognitive decline [58]. This alternative vascular hypothesis is supported by reports of cerebrovascular reactivity deficits in the posterior cingulate gyrus in older adults with vascular disease [59]. Further support for the vascular hypothesis comes from studies showing that cerebral hypoperfusion predicts cognitive decline and dementia independent of neuronal metabolic deficits measured by FDG-PET [6, 8]. The present findings offer fresh insight into this debate since posterior cingulate CBF and volume differences showed independent statistical effects on global/memory impairment, potentially suggesting distinct vascular and neurodegenerative effects on cognitive impairment in this region. The fact that cingulum white matter integrity mediated the effects of posterior cingulate hypoperfusion on global/memory impairment further supports the concept that hypoperfusion in this region may represent a vascular mechanism of brain injury.

Findings from additional exploratory analyses in both *APOE4* carriers and *APOE3* homozygotes suggest a pattern of frontal and inferior parietal hypoperfusion that contributes to deficits in attention and executive function both directly, independent of structural changes, and through decreased integrity of the superior longitudinal fasciculus white matter tract that connects these two regions. However, indirect mediation effects in these regions did not reach statistical significance, preventing a definitive conclusion from being drawn regarding independent versus mediating effects of CBF and white matter integrity on attention/executive impairment. Findings in the left inferior frontal gyrus also suggest hypoperfusion in this region may contribute to language impairment, independent of regional volume. Thus, hypoperfusion in other cortical regions outside the posterior cingulate may be less specific to *APOE4*, and could contribute to non-amnestic cognitive impairment either by directly impairing neurocognitive function or through its relationship with white matter injury in connected tracts.

We observed slightly increased CBF and regional grey matter volume within the posterior cingulate cortex in cognitively unimpaired *APOE4* carriers relative to cognitively unimpaired *APOE3/3* homozygotes. This contrasts with the *APOE4*-specific decreases in CBF and volume observed within the posterior cingulate region in those with cognitive impairment. However, these differences did not survive multiple comparison correction and are of unclear clinical significance. It is possible slight increases in CBF and grey matter volume in this region in cognitively unimpaired *APOE4* carriers represent early pathologic or compensatory changes related to genetic risk for cognitive decline. It is also possible that higher baseline CBF and volume in this susceptibility region may help to maintain normal cognition despite the presence of *APOE4*. Further insight into these questions may be gleaned from longitudinal and predictive studies examining whether the observed increases in posterior cingulate CBF and volume are longstanding or represent compensatory or pathological increases that may differentially predict future cognitive decline.

The strengths of the present study include the use of multimodal MRI to investigate regional CBF and grey and white matter structural integrity in *APOE4* carriers and *APOE3* homozygotes with and without cognitive impairment in three cognitive ability domains, as well as the mediation analysis identifying direct, independent, and indirectly mediated effects of regional CBF on cognitive impairment. Study limitations include the cross-sectional design, limiting causal inference, and the inability to study white matter hypoperfusion directly with pCASL-MRI. The sample size was also relatively small, limiting statistical power. Further longitudinal mediation studies may clarify the relative timing and predictive value of changes in regional CBF and corresponding structural integrity, yielding additional insights into the independent and interactive effects of vascular and neurodegenerative processes driving cognitive decline in *APOE4* carriers at genetic risk for dementia.

## Data Availability

The anonymous data that support the findings of this study are available upon reasonable request from the corresponding author, DAN, through appropriate data sharing protocols.

## References

[1] Gorelick PB, Scuteri A, Black SE, et al. Vascular contributions to cognitive impairment and dementia: a statement for healthcare professionals from the American Heart Association/American Stroke Association. Stroke. 2011;42(9):2672–713. 21778438 10.1161/STR.0b013e3182299496 PMC3778669

[2] Zlokovic BV, Gottesman RF, Bernstein KE, et al. Vascular contributions to cognitive impairment and dementia (VCID): a report from the 2018 National Heart, Lung, and Blood Institute and National Institute of Neurological Disorders and Stroke Workshop. Alzheimers Dement. 2020;16(12):1714–33. 33030307 10.1002/alz.12157

[3] Dounavi ME, Low A, McKiernan EF, et al. Evidence of cerebral hemodynamic dysregulation in middle-aged APOE epsilon4 carriers: The PREVENT-Dementia study. J Cereb Blood Flow Metab. 2021;41(11):2844–55. 34078163 10.1177/0271678X211020863 PMC8543665

[4] Filippini N, Ebmeier KP, MacIntosh BJ, et al. Differential effects of the APOE genotype on brain function across the lifespan. Neuroimage. 2011;54(1):602–10. 20705142 10.1016/j.neuroimage.2010.08.009

[5] Iadecola C. The neurovascular unit coming of age: a journey through neurovascular coupling in health and disease. Neuron. 2017;96(1):17–42. 28957666 10.1016/j.neuron.2017.07.030 PMC5657612

[6] Iturria-Medina Y, Sotero RC, Toussaint PJ, Mateos-Perez JM, Evans AC. Alzheimer’s disease Neuroimaging I. Early role of vascular dysregulation on late-onset Alzheimer’s disease based on multifactorial data-driven analysis. Nat Commun. 2016;7:11934. 27327500 10.1038/ncomms11934 PMC4919512

[7] Wolters FJ, Zonneveld HI, Hofman A, et al. Cerebral perfusion and the risk of dementia: a population-based study. Circulation. 2017;136(8):719–28. 28588075 10.1161/CIRCULATIONAHA.117.027448

[8] Yew B, Nation DA. Alzheimer’s disease neuroimaging I. Cerebrovascular resistance: effects on cognitive decline, cortical atrophy, and progression to dementia. Brain. 2017;140(7):1987–2001. 28575149 10.1093/brain/awx112 PMC6059092

[9] Corder EH, Saunders AM, Strittmatter WJ, et al. Gene dose of apolipoprotein E type 4 allele and the risk of Alzheimer’s disease in late onset families. Science. 1993;261(5123):921–3. 8346443 10.1126/science.8346443

[10] Yamazaki Y, Zhao N, Caulfield TR, Liu CC, Bu G. Apolipoprotein E and Alzheimer disease: pathobiology and targeting strategies. Nat Rev Neurol. 2019;15(9):501–18. 31367008 10.1038/s41582-019-0228-7 PMC7055192

[11] Schmidt H, Freudenberger P, Seiler S, Schmidt R. Genetics of subcortical vascular dementia. Exp Gerontol. 2012;47(11):873–7. 22735669 10.1016/j.exger.2012.06.003 PMC3490100

[12] Bell RD, Winkler EA, Singh I, et al. Apolipoprotein E controls cerebrovascular integrity via cyclophilin A. Nature. 2012;485(7399):512–6. 22622580 10.1038/nature11087 PMC4047116

[13] Koizumi K, Hattori Y, Ahn SJ, et al. Apoepsilon4 disrupts neurovascular regulation and undermines white matter integrity and cognitive function. Nat Commun. 2018;9(1):3816. 30232327 10.1038/s41467-018-06301-2 PMC6145902

[14] Dounavi ME, Mak E, Swann P, et al. Differential association of cerebral blood flow and anisocytosis in APOE epsilon4 carriers at midlife. J Cereb Blood Flow Metab. 2023;43(10):1672–84. 37132287 10.1177/0271678X231173587 PMC10581239

[15] McKiernan EF, Mak E, Dounavi ME, et al. Regional hyperperfusion in cognitively normal APOE epsilon4 allele carriers in mid-life: analysis of ASL pilot data from the PREVENT-Dementia cohort. J Neurol Neurosurg Psychiatry. 2020;91(8):861–6. 32586852 10.1136/jnnp-2020-322924

[16] Bangen KJ, Restom K, Liu TT, et al. Assessment of Alzheimer’s disease risk with functional magnetic resonance imaging: an arterial spin labeling study. J Alzheimers Dis. 2012;31(s3):S59-74. 22531427 10.3233/JAD-2012-120292 PMC3443702

[17] Wierenga CE, Clark LR, Dev SI, et al. Interaction of age and APOE genotype on cerebral blood flow at rest. J Alzheimers Dis. 2013;34(4):921–35. 23302659 10.3233/JAD-121897 PMC4124882

[18] Zlatar ZZ, Bischoff-Grethe A, Hays CC, et al. Higher brain perfusion may not support memory functions in cognitively normal carriers of the ApoE epsilon4 allele compared to non-carriers. Front Aging Neurosci. 2016;8:151. 27445794 10.3389/fnagi.2016.00151 PMC4919360

[19] Memel M, Staffaroni AM, Cobigo Y, et al. APOE moderates the effect of hippocampal blood flow on memory pattern separation in clinically normal older adults. Hippocampus. 2021;31(8):845–57. 33835624 10.1002/hipo.23327 PMC8295213

[20] Hays CC, Zlatar ZZ, Meloy MJ, et al. Anterior cingulate structure and perfusion is associated with cerebrospinal fluid tau among cognitively normal older adult APOEvarepsilon4 Carriers. J Alzheimers Dis. 2020;73(1):87–101. 31743999 10.3233/JAD-190504 PMC7310575

[21] Kim SM, Kim MJ, Rhee HY, et al. Regional cerebral perfusion in patients with Alzheimer’s disease and mild cognitive impairment: effect of APOE epsilon4 allele. Neuroradiology. 2013;55(1):25–34. 22828738 10.1007/s00234-012-1077-x

[22] Michels L, Warnock G, Buck A, et al. Arterial spin labeling imaging reveals widespread and Abeta-independent reductions in cerebral blood flow in elderly apolipoprotein epsilon-4 carriers. J Cereb Blood Flow Metab. 2016;36(3):581–95. 26661143 10.1177/0271678X15605847 PMC4794091

[23] Hays CC, Zlatar ZZ, Meloy MJ, et al. Interaction of APOE, cerebral blood flow, and cortical thickness in the entorhinal cortex predicts memory decline. Brain Imaging Behav. 2020;14(2):369–82. 32048144 10.1007/s11682-019-00245-x PMC7165062

[24] Wierenga CE, Dev SI, Shin DD, et al. Effect of mild cognitive impairment and APOE genotype on resting cerebral blood flow and its association with cognition. J Cereb Blood Flow Metab. 2012;32(8):1589–99. 22549621 10.1038/jcbfm.2012.58 PMC3421098

[25] Luckhaus C, Cohnen M, Fluss MO, et al. The relation of regional cerebral perfusion and atrophy in mild cognitive impairment (MCI) and early Alzheimer’s dementia. Psychiatry Res. 2010;183(1):44–51. 20541374 10.1016/j.pscychresns.2010.04.003

[26] Coupe P, Yger P, Prima S, Hellier P, Kervrann C, Barillot C. An optimized blockwise nonlocal means denoising filter for 3-D magnetic resonance images. IEEE Trans Med Imaging. 2008;27(4):425–41. 18390341 10.1109/TMI.2007.906087 PMC2881565

[27] Tustison NJ, Avants BB, Cook PA, et al. N4ITK: improved N3 bias correction. IEEE Trans Med Imaging. 2010;29(6):1310–20. 20378467 10.1109/TMI.2010.2046908 PMC3071855

[28] Avants BB, Tustison NJ, Song G, Cook PA, Klein A, Gee JC. A reproducible evaluation of ANTs similarity metric performance in brain image registration. Neuroimage. 2011;54(3):2033–44. 20851191 10.1016/j.neuroimage.2010.09.025 PMC3065962

[29] Avants BB, Epstein CL, Grossman M, Gee JC. Symmetric diffeomorphic image registration with cross-correlation: evaluating automated labeling of elderly and neurodegenerative brain. Med Image Anal. 2008;12(1):26–41. 17659998 10.1016/j.media.2007.06.004 PMC2276735

[30] Zhang Y, Brady M, Smith S. Segmentation of brain MR images through a hidden Markov random field model and the expectation-maximization algorithm. IEEE Trans Med Imaging. 2001;20(1):45–57. 11293691 10.1109/42.906424

[31] Wang Z, Aguirre GK, Rao H, et al. Empirical optimization of ASL data analysis using an ASL data processing toolbox: ASLtbx. Magn Reson Imaging. 2008;26(2):261–9. 17826940 10.1016/j.mri.2007.07.003 PMC2268990

[32] Wang Z. Improving cerebral blood flow quantification for arterial spin labeled perfusion MRI by removing residual motion artifacts and global signal fluctuations. Magn Reson Imaging. 2012;30(10):1409–15. 22789842 10.1016/j.mri.2012.05.004 PMC3482282

[33] Nation DA, Wierenga CE, Clark LR, et al. Cortical and subcortical cerebrovascular resistance index in mild cognitive impairment and Alzheimer’s disease. J Alzheimers Dis. 2013;36(4):689–98. 23666173 10.3233/JAD-130086 PMC4089500

[34] Clark LR, Nation DA, Wierenga CE, et al. Elevated cerebrovascular resistance index is associated with cognitive dysfunction in the very-old. Alzheimers Res Ther. 2015;7(1):3. 27391477 10.1186/s13195-014-0080-3 PMC4942967

[35] Petr J, Mutsaerts H, De Vita E, et al. Effects of systematic partial volume errors on the estimation of gray matter cerebral blood flow with arterial spin labeling MRI. MAGMA. 2018;31(6):725–34. 29916058 10.1007/s10334-018-0691-y

[36] Veraart J, Novikov DS, Christiaens D, Ades-Aron B, Sijbers J, Fieremans E. Denoising of diffusion MRI using random matrix theory. Neuroimage. 2016;142:394–406. 27523449 10.1016/j.neuroimage.2016.08.016 PMC5159209

[37] Smith SM. Fast robust automated brain extraction. Hum Brain Mapp. 2002;17(3):143–55. 12391568 10.1002/hbm.10062 PMC6871816

[38] Batchelor PG, Moakher M, Atkinson D, Calamante F, Connelly A. A rigorous framework for diffusion tensor calculus. Magn Reson Med. 2005;53(1):221–5. 15690523 10.1002/mrm.20334

[39] Pajevic S, Pierpaoli C. Color schemes to represent the orientation of anisotropic tissues from diffusion tensor data: application to white matter fiber tract mapping in the human brain. Magn Reson Med. 1999;42(3):526–40. 10467297

[40] Theaud G, Houde J-C, Boré A, Rheault F, Morency F, Descoteaux M. TractoFlow: a robust, efficient and reproducible diffusion MRI pipeline leveraging Nextflow & Singularity. Neuroimage. 2020;218:116889. 32447016 10.1016/j.neuroimage.2020.116889

[41] Di Tommaso P, Chatzou M, Floden EW, Barja PP, Palumbo E, Notredame C. Nextflow enables reproducible computational workflows. Nat Biotechnol. 2017;35(4):316–9. 28398311 10.1038/nbt.3820

[42] Kurtzer GM, Sochat V, Bauer MW. Singularity: scientific containers for mobility of compute. PLoS ONE. 2017;12(5):e0177459. 28494014 10.1371/journal.pone.0177459 PMC5426675

[43] Jenkinson M, Beckmann CF, Behrens TE, Woolrich MW, Smith SM. FSL Neuroimage. 2012;62(2):782–90. 21979382 10.1016/j.neuroimage.2011.09.015

[44] Tournier JD, Smith R, Raffelt D, et al. MRtrix3: a fast, flexible and open software framework for medical image processing and visualisation. Neuroimage. 2019;202:116137. 31473352 10.1016/j.neuroimage.2019.116137

[45] Garyfallidis E, Brett M, Amirbekian B, et al. Dipy, a library for the analysis of diffusion MRI data. Front Neuroinform. 2014;8:8. 24600385 10.3389/fninf.2014.00008 PMC3931231

[46] Ossendorf M, Prellwitz W. Rapid and easy apolipoprotein E genotying using an improved PCR-RFLP technique. Qiagen News. 2000;1:11–3.

[47] Bondi MW, Edmonds EC, Jak AJ, et al. Neuropsychological criteria for mild cognitive impairment improves diagnostic precision, biomarker associations, and progression rates. J Alzheimers Dis. 2014;42(1):275–89. 24844687 10.3233/JAD-140276 PMC4133291

[48] Bangen KJ, Nation DA, Delano-Wood L, et al. Aggregate effects of vascular risk factors on cerebrovascular changes in autopsy-confirmed Alzheimer’s disease. Alzheimers Dement. 2015;11(4):394-403e391. 25022538 10.1016/j.jalz.2013.12.025 PMC4287462

[49] Nation DA, Delano-Wood L, Bangen KJ, et al. Antemortem pulse pressure elevation predicts cerebrovascular disease in autopsy-confirmed Alzheimer’s disease. J Alzheimers Dis. 2012;30(3):595–603. 22451309 10.3233/JAD-2012-111697 PMC3370943

[50] Chandio BQ, Risacher SL, Pestilli F, et al. Bundle analytics, a computational framework for investigating the shapes and profiles of brain pathways across populations. Sci Rep. 2020;10(1):17149. 33051471 10.1038/s41598-020-74054-4 PMC7555507

[51] Swinford CG, Risacher SL, Wu YC, et al. Altered cerebral blood flow in older adults with Alzheimer’s disease: a systematic review. Brain Imaging Behav. 2023;17(2):223–56. 36484922 10.1007/s11682-022-00750-6 PMC10117447

[52] Zhang H, Wang Y, Lyu D, et al. Cerebral blood flow in mild cognitive impairment and Alzheimer’s disease: a systematic review and meta-analysis. Ageing Res Rev. 2021;71:101450. 34419673 10.1016/j.arr.2021.101450

[53] Chen X, Chen L, Lin G, et al. White matter damage as a consequence of vascular dysfunction in a spontaneous mouse model of chronic mild chronic hypoperfusion with eNOS deficiency. Mol Psychiatry. 2022;27(11):4754–69. 35948662 10.1038/s41380-022-01701-9 PMC9734049

[54] Alber J, Alladi S, Bae HJ, et al. White matter hyperintensities in vascular contributions to cognitive impairment and dementia (VCID): knowledge gaps and opportunities. Alzheimers Dement (N Y). 2019;5:107–17. 31011621 10.1016/j.trci.2019.02.001 PMC6461571

[55] Liu CC, Liu CC, Kanekiyo T, Xu H, Bu G. Apolipoprotein E and Alzheimer disease: risk, mechanisms and therapy. Nat Rev Neurol. 2013;9(2):106–18. 23296339 10.1038/nrneurol.2012.263 PMC3726719

[56] Dolui S, Li Z, Nasrallah IM, Detre JA, Wolk DA. Arterial spin labeling versus (18)F-FDG-PET to identify mild cognitive impairment. Neuroimage Clin. 2020;25:102146. 31931403 10.1016/j.nicl.2019.102146 PMC6957781

[57] Musiek ES, Chen Y, Korczykowski M, et al. Direct comparison of fluorodeoxyglucose positron emission tomography and arterial spin labeling magnetic resonance imaging in Alzheimer’s disease. Alzheimers Dement. 2012;8(1):51–9. 22018493 10.1016/j.jalz.2011.06.003 PMC3264701

[58] Sweeney MD, Montagne A, Sagare AP, et al. Vascular dysfunction—the disregarded partner of Alzheimer’s disease. Alzheimers Dement. 2019;15(1):158–67. 30642436 10.1016/j.jalz.2018.07.222 PMC6338083

[59] Haight TJ, Bryan RN, Erus G, et al. Vascular risk factors, cerebrovascular reactivity, and the default-mode brain network. Neuroimage. 2015;115:7–16. 25917517 10.1016/j.neuroimage.2015.04.039 PMC4469180

